# Effects of Cement Paste Enhanced with Iron-Based Magnetic Particles on an Embedded Small Resonator Antenna

**DOI:** 10.1038/s41598-017-15289-6

**Published:** 2017-11-09

**Authors:** Yee Loon Sum, Vanessa Rheinheimer, Boon Hee Soong, Paulo J. M. Monteiro

**Affiliations:** 1 Nanyang Technological University, Electrical and Electronic Engineering, Singapore, 639798 Singapore; 2 Berkeley Education Alliance for Research in Singapore, Singapore, 138602 Singapore; 3 University of California, Berkeley, Civil Engineering, California, 94720 USA

## Abstract

Small resonator antennas, such as metaresonator antennas, have narrow bandwidths, which limits their effective range of frequencies. When they are used as embedded antennas in building materials, their performance is affected more than other types of antennas, as typical building materials have a shielding effectiveness (SE) of 80 dB to 100 dB. Adding magnetic and/or metallic particles to cement mixes changes the properties of the concrete, which can improve the performance of antennas. Specifically, enhancing a cement paste with iron-based magnetic particles improves the bandwidth and S_11_ of embedded antennas. This report investigates the impact of two different iron-based magnetic particle sizes (micro- and nanosized particles) to determine the effects that they have on the S_11_ and S_21_ characteristics of the metaresonator antenna array embedded in enhanced cement pastes. Results show that compared to cement paste only sample, cement paste with micro-sized iron-based magnetic particles had the greatest improvement of performance of a metaresonator antenna array in terms of a small shift in the resonance frequency and an increase of bandwidth. Particularly for a cement paste enhanced with micro-sized iron (III) oxide particles, the S_21_ curve was improved over the cement paste only sample by as much as 10 dB.

## Introduction

Wireless communications are essential in buildings for many reasons, including internet of things (IOT) applications. However, the materials used in buildings do not enable good propagation of wireless signals within and in and out of buildings. This deficiency is a reality for antennas in air and for antennas embedded in building materials. With increasing interest to use such embedded antennas in building materials for concrete health monitoring^1,2^, wireless powering of embedded sensors^3^ and radio-frequency identification (RFID) applications^4^, investigations have been conducted to study the effects of building materials on electromagnetic (EM) propagation. It is known that common building materials, such as concrete, gypsum and plaster, have high shielding effectiveness (SE) values. Direct measurements^5,6^, and analysis using finite-difference time-domain (FDTD)^7^, and method of moments (MOM)^8^ techniques show that building materials have more than 80 dB to 100 dB of SE. One way to improve the performance of concrete is by adding magnetic and/or conductive particles into the cement mix. Historically, the addition of particles in concrete focused on the need to dispose of industry residues (an environmental aspect), and existing studies of this composite material with additions of magnetic and/or conductive particles have been restricted to concrete performance and durability^9–12^, not applying their use for enhancing the EM properties of the concrete structure for embedded antennas to date. Furthermore, the use of conductive concrete for EM applications to date has been focusing on improving the SE of concrete. Previous work developed conductive concrete for EM applications using carbon fibers in polymer concretes^13^, and electro conductive concrete^14^ with mortar blocks for frequencies from 30 MHz to 5 GHz to improve the SE of materials. The SE achieved are 65 dB at 500 MHz, 75 dB at 1 GHz, and 95 dB at 1.5 GHz. Other researchers used different concrete mixtures with steel and carbon fibers, and different grades of carbon powder, achieving an SE of 52 dB at 1 GHz^15^, or using concrete mixtures with carbon fibers attaining a SE of approximately 20 dB to 30 dB between the frequencies of 1 GHz and 2 GHz^16^. In this work, we proposed adding iron-based magnetic particles in cement paste to improve the EM properties of concrete for embedded antennas. Two types of particles of two different sizes were added to a cement paste with an embedded metaresonator antenna array to simulate embedded wireless applications. As a result of an improvement in the electrical properties of the heterogeneous medium, we observed for all samples 1) an increase in the bandwidth of the embedded antenna compared to the antenna in air and 2) a shift in the resonance frequency that was smaller than that of an antenna in cement paste alone. In the analysis of the effects that different sizes of iron-based particles have on the antennas, it was observed that the cement paste enhanced with the addition of micro-sized particles while increasing the bandwidth was able to maintain the general shape of the *S*_11_ curve of the antenna compared to other samples and the control. In addition, for the intended WiFi spectrum, cement paste samples containing micro-sized iron (III) oxide improved the transmission coefficient of the antenna by as much as 10 dB.

## Results

### Effects on reflection coefficient

**Figure 1:**
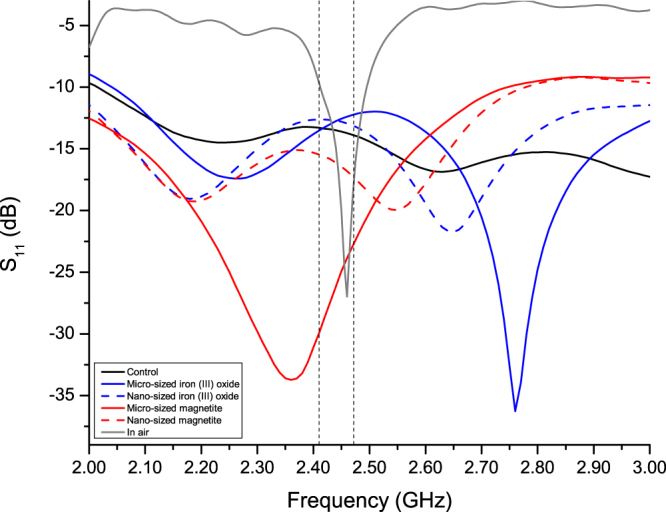
Comparison of *S*_11_ of antenna array in air, and other cement paste enhanced particles from 2 to 3 GHz.

Figure 1 shows the measured reflection coefficient, *S*_11_, of the antenna in cement paste only (solid black line) and in other cement pastes enhanced with particles. As there were slight deviations in the performance of each antenna, the *S*_11_ of one sample antenna measured in air medium (solid gray line) is shown as a reference. The frequency range (between the two vertical dashed lines) represents the intended WiFi spectrum. When an antenna was embedded in cement paste only (the solid black line), the behavior of the antenna was changed significantly. At the designed frequency of 2.442 GHz, the *S*_11_ moved by −13 dB. Within the 2 GHz to 3 GHz bandwidth, there was no noticeable resonance, although in general, the *S*_11_ value is lower than −10 dB. This finding demonstrates that the impedance of the antenna was not well-matched throughout this bandwidth. To improve the performance of an embedded antenna, four types of iron-based magnetic particles were added to the cement paste mix: 1) micro-sized magnetite (solid red line), 2) nanosized magnetite (dashed red line), 3) micro-sized iron (III) oxide (solid blue line), and 4) nanosized iron (III) oxide (dashed blue line). Table 1 summaries the effects that the different particles had on the antenna. The change in *S*_11_ (in dB) and frequency shifts (in percentage, where a positive value indicates a shifting to higher frequencies, and a negative value indicate a shifting to lower frequencies) are compared to the measurement of the antenna in air. The change in *S*_11_ is taken as a decrease of the lowest *S*_11_ value within the 2 to 3 GHz frequency range of the antenna measured before and after embedding into the cement paste.

**Table 1:** Effects of media on *S*_11_ and resonance frequency.

Sample type	Change in *S*_11_ (dB)	Frequency shift (%)
Cement paste only (Control)	−2.01	23.20
Cement paste with micro-sized magnetite particles	−17.26	−3.87
Cement paste with nanosized magnetite particles	−6.19	3.66
Cement paste with micro-sized iron oxide particles	−19.28	12.20
Cement paste with nanosized iron oxide particles	−9.07	8.35

For the cement paste only sample (the control), the *S*_11_ value decreased by 2.01 dB compared to the measurement for the antenna in air. As there was no noticeable dip in the *S*_11_ within the 2 to 3 GHz range, the lowest point of the *S*_11_ at 3 GHz was taken as the resonance frequency for comparison purposes. For cement paste enhanced with nanosized particles, it was observed that both magnetite and iron (III) oxide containing samples cause the *S*_11_ values to decrease by 6.19 dB and 9.07 dB, respectively. For cement paste enhanced with micro-sized particles, the magnetite and iron (III) oxide containing samples caused the *S*_11_ values to decrease by 17.26 dB and 19.28 dB, respectively. Comparing the samples with different types of particles, the samples containing iron (III) oxide particles showed a decrease in the *S*_11_ larger than samples containing magnetite particles, promoting a better performance of the embedded antenna. Comparing the samples with different particle sizes, the addition of micro-sized particles was able to lower the *S*_11_ of the embedded antenna to be even better than the sample reference antenna measured in air. This shows that when an antenna was embedded into a cement paste containing micro-sized magnetic iron-based particles, the *S*_11_ value was significantly better than just the antenna in air. Conversely, although cement pastes with nanosized magnetic iron-based could lower the *S*_11_ of the antennas, they were not able to significantly improve this performance such that the general results were not better than just the antenna in air. In all samples, the *S*_11_ was reduced by less than −10 dB within the intended WiFi bandwidth. A reading of −10 dB or less indicates a sufficient performance of the antenna where at least 90% of the input power is delivered to the antenna, and 10% is reflected.

Observing the shift in resonance frequency, assuming the resonance frequency of the control sample at 3 GHz (lowest value within the test spectrum), the shift in frequency is 23.2%. For the cement paste with magnetite particles sample, the shifts in frequency is −3.87% and 3.66% for micro-sized and nano-sized particles respectively. Compared with the cement paste with iron (III) oxide particles samples, the shift is 12.20% and 8.35% for the micro-sized and nanosized particles respectively. In general, other than the cement paste with micro-sized magnetite particles, the other three cement paste samples with particles had their resonance frequencies shifted to higher frequencies. In terms of the magnitude of shifting, the samples with iron (III) oxide had their resonance frequencies shifted more than those of the magnetite samples, with the cement paste sample with micro-sized iron (III) oxide shifting towards higher frequency the most. A small shift in frequency shows that the material had little detuning effect on the antenna.

When observing the shape of the *S*_11_, it can be noted that the cement paste samples with micro-sized particles maintained the general shape of the *S*_11_ curves with a single distinct resonance frequency. The cement pastes with nanosized particles exhibited two resonance frequencies, altering the single resonance frequency shape of the antenna. In terms of bandwidth, both cement pastes with micro-sized particles increased the bandwidth, with magnetite particles having a larger effect than iron (III) oxide. For the nanosized particles, the double dip shape also had an increased bandwidth when compared to the bandwidth of the reference antenna in air.

From the observations of the *S*_11_ of the different samples, adding iron-based magnetic particles had a positive effect on the embedded antenna. The cement paste samples with micro-sized particles had a better effect than the cement pastes with nanosized particles on the antennas in terms of the decrease in *S*_11_, bandwidth, and the shape of the *S*_11_ curve.

### Effects on transmission coefficient

**Figure 2:**
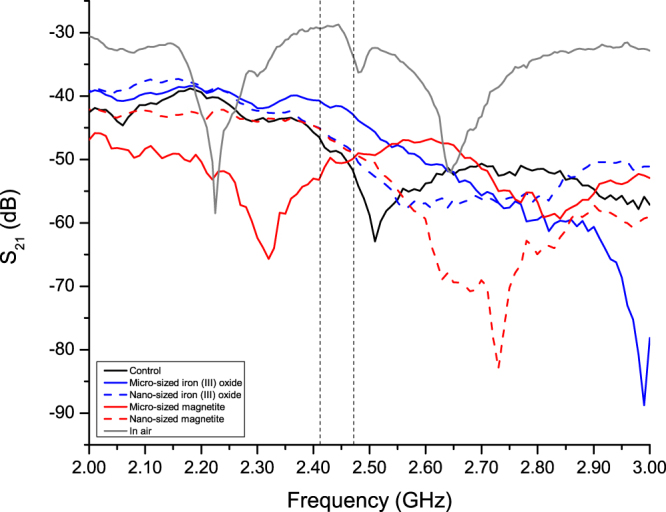
Comparison of *S*_21_ of antenna array in air, and other cement paste enhanced with particles from 2 to 3 GHz.

Figure 2 shows the measured transmission coefficients of the samples taken between 2 and 3 GHz. The frequencies between the two vertical dotted lines indicates the WiFi spectrum. A reference measurement of the sample antenna used (the solid gray line) is shown to indicate the performance of the antenna in air (not embedded). At the intended frequency range of WiFi, the *S*_21_ has a range of −30 dB to −37 dB. When the antenna was placed in a cement paste only (the solid black line), the *S*_21_ was reduced by approximately 16 to 20 dB. This shows that the amount of EM radiation that can pass through into the cement paste was significantly reduced. Focusing on the WiFi spectrum (within the 2 vertical dashed lines), when the antenna was placed in a cement paste with iron-based magnetic particles, most of the *S*_21_ curves improved except from 2.41 GHz to 2.46 GHz for the cement paste sample with micro-sized magnetite particles. Both cement pastes with nanosized particles had the same behavior in this range. For cement pastes with iron (III) oxide particles, the improvement of the *S*_21_ curve was the largest. From Table 2, which shows a summary of the *S*_21_ within the WiFi spectrum, the cement paste with micro-sized magnetite produced the least improvement, with certain frequencies performing worse than the control sample (cement paste only). On the other hand, the cement pastes with iron (III) oxide sample presented the most improvement (up to 10.33 dB), observed at Channel 13 (2.472 GHz) of the WiFi band. This finding shows that by enhancing the cement paste with iron-based magnetic particles, the amount of EM radiation passing through the material increases compared to cement paste alone.

**Table 2:** Effects of media on *S*_21_ within WiFi spectrum.

*Sample type	*S*_21_ (dB)		Change in *S*_21_ (dB)	
	Channel 1	Channel 13	Channel 1	Channel 13
Cement paste only (Control)	−46.47	−54.21	—	—
Cement paste with micro-sized magnetite particles	−53.23	−49.46	−6.76	4.75
Cement paste with nanosized magnetite particles	−45.1	−49.35	1.37	4.75
Cement paste with micro-sized iron oxide particles	−40.49	−43.88	5.98	10.33
Cement paste with nanosized iron oxide particles	−45.2	−49.52	1.27	4.69

For frequencies lower than Channel 1 of WiFi (the left hand region of the left vertical dashed line), samples containing iron (III) oxide particles showed a better effect on *S*_21_ compared to the control and samples with magnetite particles. At frequencies higher than Channel 13 of WiFi (the right hand region of the right vertical dashed line), samples with nanosized magnetite particles showed a poor effect on *S*_21_ compared to the other samples.

## Discussion

Changing the medium surrounding an antenna changes its behavior due to the different characteristic impedances. The material of the antenna form capacitors and/or inductors which are coupled to its surrounding medium. Any metallic, magnetic, and/or dielectric material in the near field affects these inductive and capacitive values. When discussing small resonant antennas, such as metaresonators, the quality factor is important, as they typically have a small bandwidth, *Bw*. The quality factor provided by the Chu-limit^17^ is given as

1 $$Q > \frac{1}{{(ka)}^{3}}+\frac{1}{ka}$$

where $k=\frac{2\pi }{\lambda }$, and *a* is the radius of the sphere enclosing the antenna.

In general, the quality factor^18^ can be written as

2 $$Q=\frac{\omega W}{{P}_{rad}}$$

where *ω* is the angular frequency, *W* is the average stored energy, and $Prad$ is the radiated power. It is beneficial that *Q* is small as *BW* is inversely proportional to *Q*, by having as little energy stored in the antenna, and increasing the power radiated. One way to decrease the *Q* and improve the *BW* is to analyze the antenna using series and parallel resonance transmission lines (TLs). If the transverse electromagnetic is regarded, TEM wave propagation on a two-wire conductor, and assuming sinusoidal steady-state, the complex propagation constant can be obtained:

3 $$\gamma =\alpha +j\beta =\sqrt{(R+j\omega L)(G+j\omega C)}$$

where *α* is the attenuation constant, $\beta ={k}_{0}\sqrt{{\mu }_{r}{\varepsilon }_{r}}$ is the phase constant, ${k}_{0}=\omega \sqrt{{\mu }_{0}{\varepsilon }_{0}}$ is the propagation constant (wave number) of a plane wave in free space, *R* is the series resistance per unit length (*ω*/*m*), *L* is the series inductance per unit length (*H*/*m*), *G* is the shunt conductance per unit length (*S*/*m*), and *C* is the shunt capacitance per unit length (*F*/*m*). From this perspective, the quality factor can be written as

4 $$Q=\frac{\beta }{2\alpha }=\frac{{k}_{0}\sqrt{{\mu }_{r}{\varepsilon }_{r}}}{2\alpha }$$

Therefore, to lower the *Q*, it is possible to load the antenna with materials that have lower relative permittivity, ${\varepsilon }_{r}$, and relative permeability, ${\mu }_{r}$. From the results obtained, the *Q* is decreased as the *BW* increased for all four samples. The cement paste containing micro-sized iron-based magnetic particles improve the *BW* of the antenna by a larger extend while maintaining the shape of the *S*_11_ curve. Among the two cement pastes with micro-sized particles samples, iron (III) oxide had a better effect on *S*_21_ for frequencies between 2 to 2.54 GHz, which suggests that more EM waves are passing into the sample. Within the intended WiFi spectrum, the cement paste with micro-sized iron (III) oxide particles allows the most EM waves to penetrate into the sample.

## Methods

**Figure 3:**
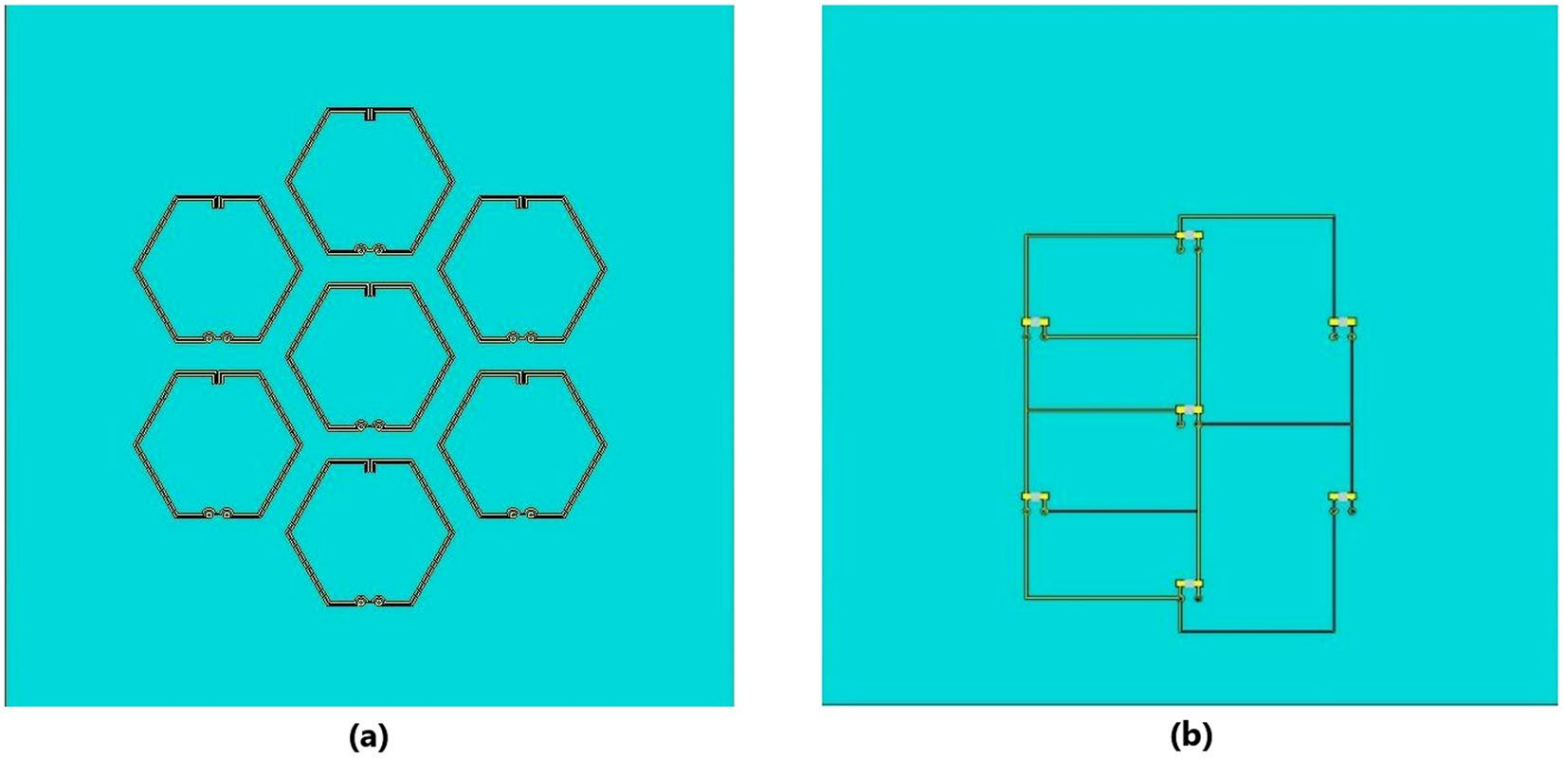
Metaresonator array antenna (**a**) Front, (**b**) Back.

**Figure 4:**
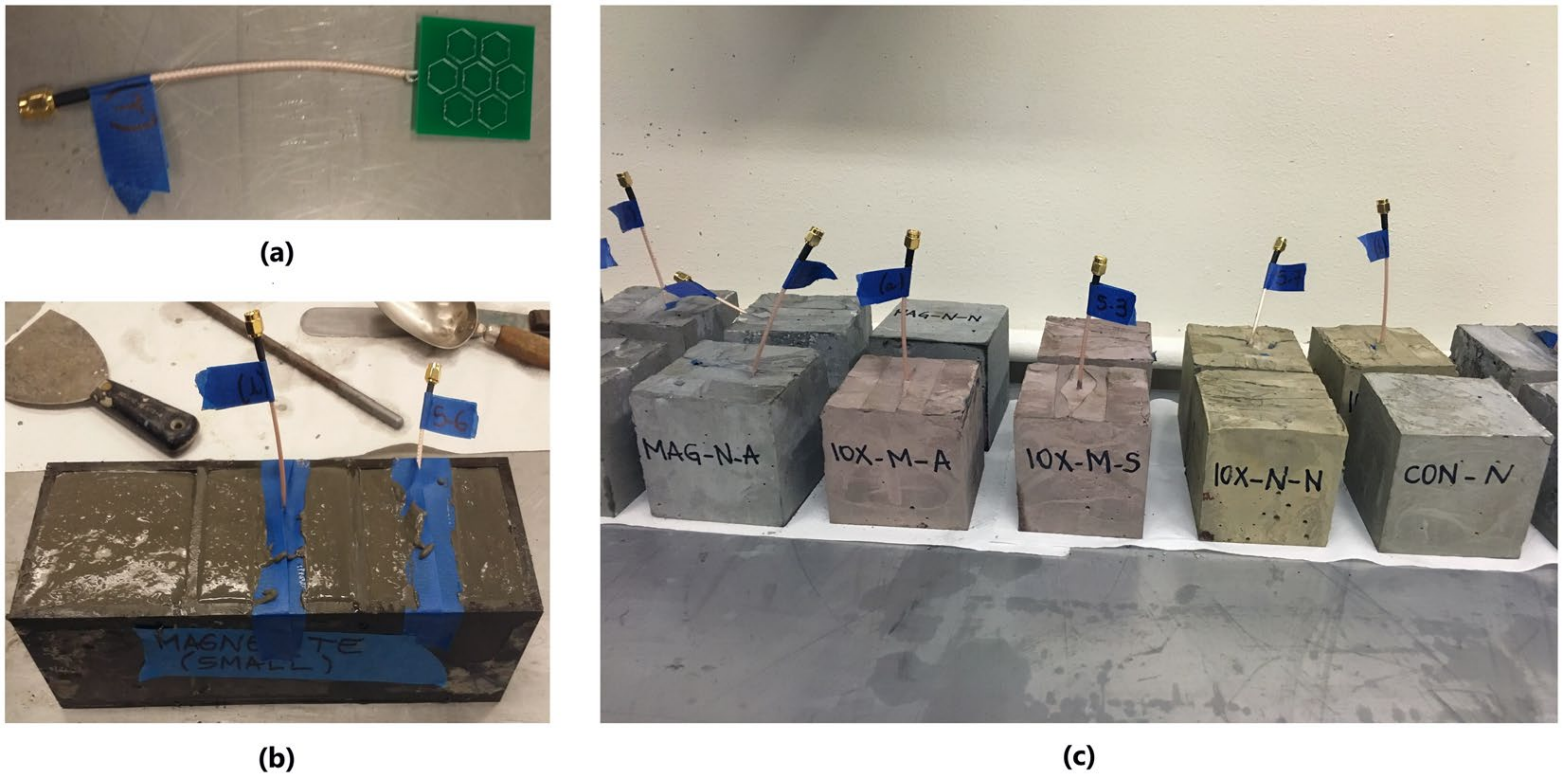
Samples of antenna in enhanced cement paste (**a**) sample metaresonator array (**b**) preparation in molds (**c**) all samples.

**Figure 5:**
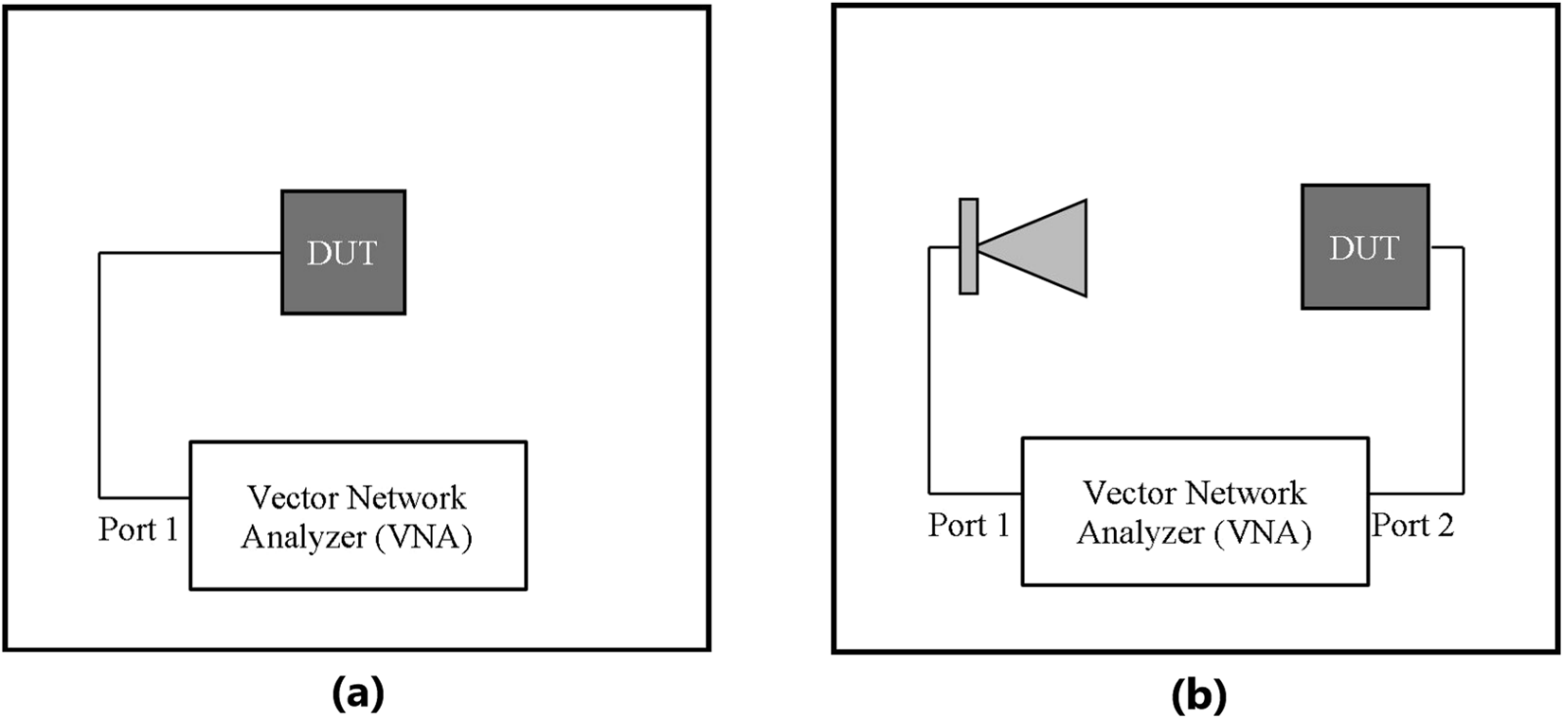
Experimental setup (**a**) measurement of *S*_11_, (**b**) measurement of *S*_21_.

Two types of particles were used: magnetite and iron (III) oxide. Since the particle size has an influence on the properties of the enhanced cement paste, two different size ranges (micro- and nanometer sized) were accessed. Details of the particles used are listed in Table 3. To simulate the embedded antenna in cement paste, metaresonator antenna arrays (see Fig. 3)^19,20^ are inserted in the center of the samples. Three cubic samples of 8 × 8 × 8 cm were produced for each mix with different particles, and cement paste alone as a control, illustrated in Fig. 4b. The samples labeling followed the nomenclature of (type of particle)-(size of particle), where the type of particle can be magnetite (MAG), iron oxide (IOX), or control (CON); and the size of the particles can be micro- (M) or nanosized (N). Ordinary Portland cement was used with a water/cement ratio of 0.35 and 0.5 weight % of particles. A superplasticizer was used at 260 *ml*/100 *kg* of cement to improve the workability. Figure 4a shows the metaresonator array with coaxial radio frequency (RF) cables soldered onto the feed points of the antenna. The cables extended of the samples (see Fig. 4c) for measurements. The samples were cured for 3 days, after which the data presented in this study were acquired. While the samples were cured for 3 days, the effect of longer curing period has been studied, and the results are given in the Supplementary Material. A N5242A PNA-X network analyzer was used to measure the *S*_11_ of the samples via a coaxial cable, and a reference antenna was connected to another port to measure the *S*_21_ parameter. The experimental setups are shown in Fig. 5. *S*_11_ is measured with antenna embedded in cement paste samples connected to port 1 (see Fig. 5a), while *S*_21_ is measured with the reference antenna in air connected to port 1 and the antenna in cement paste samples connected to port 2 (see Fig. 5b). The data plot of the *S*_11_ was captured and the frequency range of 2 to 3 GHz are displayed where the resonance frequency can be seen, together with the bandwidth and *S*_11_ values. The *S*_21_ measurement compares the amount of RF energy that can pass into the materials. As the metaresonator arrays were simulated and optimized in the CST software for air, the *S*_11_ and *S*_21_ characteristics of the embedded metaresonators were shifted away from the resonant frequency of channel 7 (2.442 GHz). This gives a good indication of the effects of the enhanced cement paste on an antenna in terms of the frequency shift. Therefore, using these two plots, a comparison to assess the effects of small resonator antennas when embedded in building materials with and without the use of particles can be performed.

**Table 3:** Types of particles, size, percentage by weight used and label.

Sample type	Size	Percentage by weight	Label
Mirco-sized magnetite	<5 $\mu m$	0.5%	MAG-M
Nanosized magnetites	5–100 $nm$	0.5%	MAG-N
Mirco-sized iron (III) oxide	<5 $\mu m$	0.5%	IOX-M
Nanosized iron (III) oxide	<50 $nm$	0.5%	IOX-N

### Data Availability

The datasets generated during and/or analyzed during the current study are available from the corresponding author upon reasonable request.

## Electronic supplementary material

Supplementary Information

## References

[1] Moosazadeh M, Kharkovsky S, Case JT, Samali B (2017). Miniaturized uwb antipodal vivaldi antenna and its application for detection of void inside concrete specimens. IEEE Antennas and Wireless Propagation Letters.

[2] Castorina G, Donato LD, Morabito AF, Isernia T, Sorbello G (2016). Analysis and design of a concrete embedded antenna for wireless monitoring applications [antenna applications corner]. IEEE Antennas and Propagation Magazine.

[3] Jiang S, Georgakopoulos SV (2012). Optimum wireless powering of sensors embedded in concrete. IEEE Transactions on Antennas and Propagation.

[4] Jeong SH, Son HW (2011). Uhf rfid tag antenna for embedded use in a concrete floor. IEEE Antennas and Wireless Propagation Letters.

[5] Micheli D, Delfini A, Santoni F, Volpini F, Marchetti M (2015). Measurement of electromagnetic field attenuation by building walls in the mobile phone and satellite navigation frequency bands. IEEE Antennas and Wireless Propagation Letters.

[6] Micheli D (2016). Electromagnetic shielding of building walls: From roman times to the present age. IEEE Antennas and Propagation Magazine.

[7] Tatematsu A, Rachidi F, Rubinstein M (2015). Analysis of electromagnetic fields inside a reinforced concrete building with layered reinforcing bar due to direct and indirect lightning strikes using the fdtd method. IEEE Transactions on Electromagnetic Compatibility.

[8] Zhao Z, Cui X, Li L, Gao C (2008). Analysis of shielding performance of reinforced concrete structures using the method of moments. IEEE Transactions on Magnetics.

[9] Blyszko J (2008). Study of mechanical properties of concrete with low concentration of magnetic nanoparticles. Journal of Non-Crystalline Solids.

[10] Guskos N (2010). Ferromagnetic resonance and compressive strength study of cement mortars containing carbon encapsulated nickel and iron nanoparticles. Rev. Adv. Mater. Sci.

[11] Guskos N (2008). Magnetic properties of the micro-silica/cement matrix with carbon-coated cobalt nanoparticles and free radical dpph. Journal of Non-Crystalline Solids.

[12] Amin MS, El-Gamal SMA, Hashem FS (2013). Effect of addition of nano-magnetite on the hydration characteristics of hardened portland cement and high slag cement pastes. Journal of Thermal Analysis and Calorimetry.

[13] Gunasekaran, M. A simplified low-cost materials approach to shielding in emc applications. In *Electromagnetic Compatibility, 1990. Seventh International Conference on*, 58–61 (IET, 1990).

[14] Tarmac. Electro conductive concrete. In 2008 IET Seminar on Electromagnetic Propagation in Structures and Buildings, 1–20 (2008).

[15] Chung D (2004). Electrically conductive cement-based materials. Advances in Cement Research.

[16] Chiou J-M, Zheng Q, Chung D (1989). Electromagnetic interference shielding by carbon fibre reinforced cement. Composites.

[17] Chu LJ (1948). Physical limitations of omni directional antennas. Journal of Applied Physics.

[18] Pozar, D. *Microwave Engineering* 4 edn (Wiley, 2011).

[19] Sum, Y. L., Rheinheimer, V., Soong, B. H. & Monterio, P. Scalable 2.45 ghz electrically small antenna design for metaresonator array. *The Journal of Engineering* (2017).

[20] Sum, Y. L., Soong, B. H. & Tseng, K. J. Metaresonator for em/rf shielding and energy harvesting. Singapore Provisional Patent 10201606290S (2016).

