# Supplementary material for: Effects of Cement Paste Enhanced with Iron-Based Magnetic Particles on an Embedded Small Resonator Antenna
Source: Sci Rep. 2017 Nov 9;7:15185. doi: 10.1038/s41598-017-15289-6 (PMC5680316; doi:10.1038/s41598-017-15289-6)
Supplement: Supplementary file 1 — Supplementary Information [file 41598_2017_15289_MOESM1_ESM.pdf]

Effects of Cement Paste Enhanced with Iron-Based Magnetic Particles on an Embedded Small Resonator Antenna

Yee Loon Sum[1,*], Vanessa Rheinheimer[2], Boon Hee Soong[1], and Paulo J.M. Monteiro[3]

[1]Nanyang Technological University, Electrical and Electronic Engineering, Singapore, 639798, Singapore
[2]Berkeley Education Alliance for Research in Singapore, Singapore, 138602, Singapore
[3]University of California, Berkeley, Civil Engineering, California, 94720, USA
[*]ylsum@ntu.edu.sg

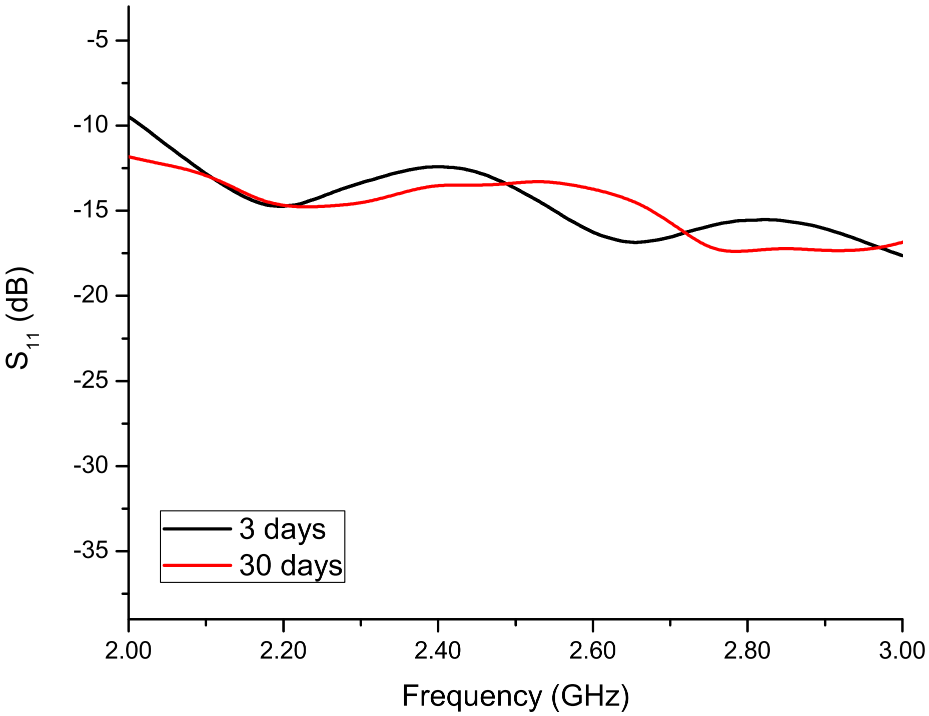

Supplementary Figure S1. Comparison of S_{11} of cement paste only (control) between 3 days and 30 days from 2 to 3 GHz

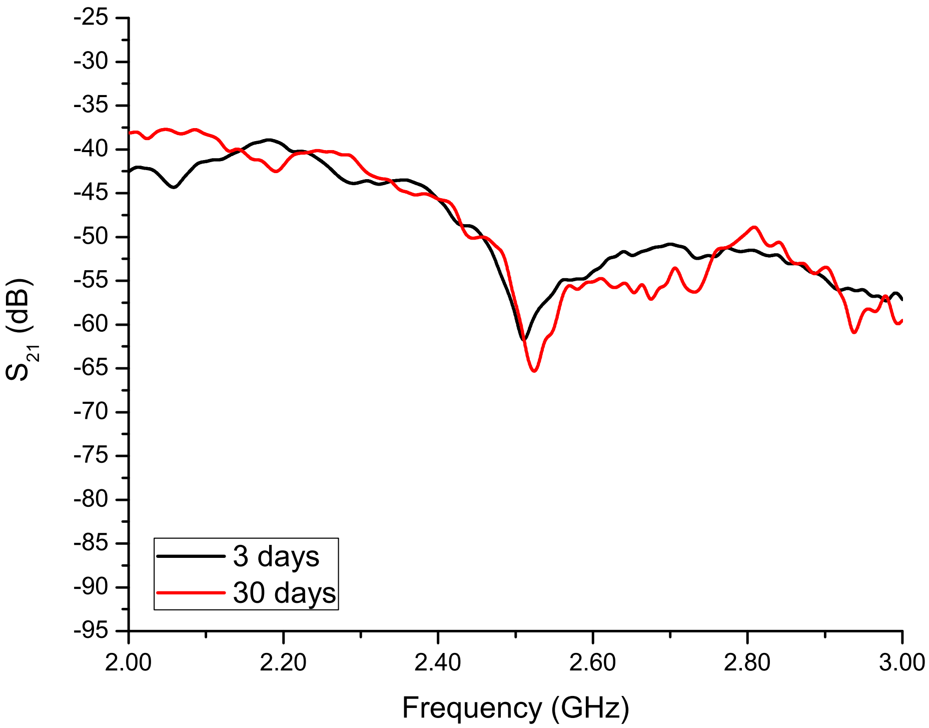

Supplementary Figure S2. Comparison of S_{21} of cement paste only (control) between 3 days and 30 days from 2 to 3 GHz

Comparison of sample between 3 days and 30 days

The comparison between the same sample of cement paste only (control) between 3 days and 30 days are shown in Supplementary Fig. S1 and Supplementary Fig. S2. Supplementary Fig. S1 shows that the S_{11} behavior do not deviate by more than 2 dB. In terms of S_{21}, Supplementary Fig. S2 also shows that the S_{21} behaviors are consistent between 3 days and 30 days. These results show that the behaviors of S_{11} and S_{21} of the antenna embedded in the samples of 3 days old and 30 days old do not deviate significantly.
